# Plasma protein biomarkers for primary graft dysfunction after lung transplantation: a single-center cohort analysis

**DOI:** 10.1038/s41598-022-20085-y

**Published:** 2022-09-27

**Authors:** Lourdes Chacon-Alberty, Rupa S. Kanchi, Shengbin Ye, Camila Hochman-Mendez, Daoud Daoud, Cristian Coarfa, Meng Li, Sandra L. Grimm, Maher Baz, Ivan Rosas, Gabriel Loor

**Affiliations:** 1 Department of Regenerative Medicine Research, Texas Heart Institute, Houston, TX USA; 2 Dan L Duncan Comprehensive Cancer Center, Baylor College of Medicine, Houston, TX USA; 3 Department of Statistics, Rice University, Houston, TX USA; 4 Division of Cardiopulmonary Transplantation and Mechanical Circulatory Support, Michael E DeBakey Department of Surgery, Baylor College of Medicine, Houston, TX USA; 5 Molecular and Cellular Biology Department, Baylor College of Medicine, Houston, TX USA; 6 Center for Precision Environment Health, Baylor College of Medicine, Houston, TX USA; 7 Department of Medicine, Baylor College of Medicine, Houston, TX USA; 8 Cardiothoracic Surgery, Texas Heart Institute, Houston, TX USA; 9 Division of Cardiopulmonary Transplantation, CHI St. Luke’s Health—Baylor St. Luke’s Medical Center, 6770 Bertner Avenue, Suite C355, Houston, TX 77030 USA

**Keywords:** Cell death and immune response, Chemokines, Innate immunity, Biomarkers, Medical research

## Abstract

The clinical use of circulating biomarkers for primary graft dysfunction (PGD) after lung transplantation has been limited. In a prospective single-center cohort, we examined the use of plasma protein biomarkers as indicators of PGD severity and duration after lung transplantation. The study comprised 40 consecutive lung transplant patients who consented to blood sample collection immediately pretransplant and at 6, 24, 48, and 72 h after lung transplant. An expert grader determined the severity and duration of PGD and scored PGD at T0 (6 h after reperfusion), T24, T48, and T72 h post-reperfusion using the 2016 ISHLT consensus guidelines. A bead-based multiplex assay was used to measure 27 plasma proteins including cytokines, growth factors, and chemokines. Enzyme-linked immunoassay was used to measure cell injury markers including M30, M65, soluble receptor of advanced glycation end-products (sRAGE), and plasminogen activator inhibitor-1 (PAI-1). A pairwise comparisons analysis was used to assess differences in protein levels between PGD severity scores (1, 2, and 3) at T0, T24, T48, and T72 h. Sensitivity and temporal analyses were used to explore the association of protein expression patterns and PGD3 at T48–72 h (the most severe, persistent form of PGD). We used the Benjamini–Hochberg method to adjust for multiple testing. Of the 40 patients, 22 (55%) had PGD3 at some point post-transplant from T0 to T72 h; 12 (30%) had PGD3 at T48–72 h. In the pairwise comparison, we identified a robust plasma protein expression signature for PGD severity. In the sensitivity analysis, using a linear model for microarray data, we found that differential perioperative expression of IP-10, MIP1B, RANTES, IL-8, IL-1Ra, G-CSF, and PDGF-BB correlated with PGD3 development at T48–72 h (FDR < 0.1 and *p* < 0.05). In the temporal analysis, using linear mixed modeling with overlap weighting, we identified unique protein patterns in patients who did or did not develop PGD3 at T48–72 h. Our findings suggest that unique inflammatory protein expression patterns may be informative of PGD severity and duration. PGD biomarker panels may improve early detection of PGD, predict its clinical course, and help monitor treatment efficacy in the current era of lung transplantation.

## Introduction

Primary graft dysfunction (PGD) is the main cause of early morbidity and mortality after lung transplantation. Although patients can be successfully treated with supportive care, there is no cure for PGD^1,2^. Furthermore, supportive care alone cannot prevent the potential for irreversible harm to the donor allograft or other end organs. Short of identifying a cure, the ability to accurately map a molecular signature for detecting PGD could enhance the overall treatment of patients. The clinical implementation of biomarkers specific to the pathogenesis of PGD would improve early detection, predict the clinical course of PGD, and, importantly, provide an objective metric to gauge the efficacy of interventions^3–5^.

The pathogenesis of PGD involves ischemia–reperfusion injury leading to inflammation, cell death, and endothelial dysfunction^6–13^. Accordingly, several studies have identified byproducts and mediators of these events as potential biomarkers associated with PGD. In a single-center study using enzyme linked immunoassays (ELISA) to detect inflammatory mediators in the plasma, Mathur et al. showed that interleukins 6, 8, and 10 (IL-6, IL-8, IL-10) and tumor necrosis factor (TNF)-α were elevated in patients with severe PGD compared with those without severe PGD^14^. In a multicenter study using a multiplex bead based assay to detect inflammatory protein mediators in the plasma, Hoffman and colleagues showed that interferon gamma-induced protein 10 (IP-10) and monocyte chemoattractant protein-1 (MCP-1) levels were higher in patients with severe PGD than in those without PGD^15^. Studies using the Lung Transplant Outcomes Group (LTOG) biorepository have validated several protein biomarkers for PGD including soluble receptor for advanced glycation end products (sRAGE), plasminogen activator inhibitor-1 (PAI-1), and MCP-1^8,16,17^.

Despite the well-founded enthusiasm in the transplant community for discovering biomarkers, they have not been adopted in clinical practice for several reasons. First, there is a lack of consensus regarding the best method for detecting biomarkers in an efficient, reliable, and cost-effective manner. Second, making consequential clinical decisions based on biomarker results is concerning because of the possibility of a false positive or negative. Finally, there is a paucity of recent studies that validate the results previously reported from observational registries, which is not surprising given the expense of collecting and analyzing these rare samples.

Thus, the current study was conducted to validate the utility of protein biomarkers for detecting the severity and duration of PGD. We used the most updated PGD grading guidelines, a contemporary cohort of lung transplant recipients, and novel statistical methods to aid in detecting a wide breadth of biomarkers.

## Methods

### Study population and design

We included all consecutive lung transplants performed at Baylor St. Luke’s Medical Center from February 25, 2018–May 30, 2019. Patients provided informed consent for data and sample collection and specimen storage. We followed our standard immunosuppression regimen in all cases including induction with basiliximab (20 mg IV), solumedrol (1000 mg IV), and mycophenolate (1000 mg IV). We gradually tapered steroids over 30 days. We used daily tacrolimus and mycophenolate mofetil for maintenance immunosuppression. Virtual cross matches were performed before transplant to confirm immune compatibility between recipient and donor.

Clinical outcomes data were entered into the Baylor-St. Luke’s Medical Center lung transplant database. All perioperative chest radiographs and blood gas results were reviewed by an expert PGD grader, and PGD scores were assigned based on strict adherence to the 2016 International Society for Heart and Lung Transplantation (ISHLT) consensus guidelines and caveats, including use of a saturation scale for patients who were extubated without arterial blood gasses^4,5^. PGD scores were determined at T0, T24, T48, and T72 h timepoints, which correspond to 6, 24, 48, and 72 h after lung reperfusion with a range of ± 6 h, except for T0 (with a range of ± 2 h). All measurements were blinded to the maximum extent possible.

### Sample collection

To ensure maximal consistency, we followed a standardized protocol for peripheral blood collection, processing, and storage. We collected peripheral blood (10 mL) in EDTA tubes at baseline (pretransplant) and 6-, 24-, 48-, and 72-h post-reperfusion and immediately transferred the samples to the Texas Heart Institute Biorepository for sample processing, storage, and biomarker analysis.

### Immunologic analyses

For biomarker analysis, blood was centrifuged; the plasma was isolated, immediately flash frozen, and stored at − 80 °C. Plasma samples were slowly thawed on ice and processed according to the manufacturer’s recommendations for multiplex bead array (Bio-Plex, Bio-Rad Laboratories, Hercules, CA, USA) (Supplementary Table 1). The plates were read with the Luminex MAGPIX with a lower limit of 100 beads per sample per analyte, and protein concentrations were analyzed using the Bio-plex Results Generator. A coefficient of variation < 20% was used as acceptance criteria. The multiplex assay was used to detect the expression levels of 27 cytokines, chemokines, and growth factors. Enzyme-linked immunoassay (ELISA) was used to detect expression levels of proteins that were not available in the multiplex assay including PAI-1, cell death markers (M30, M65), and sRAGE. All our methods were carried out in accordance with relevant guidelines and regulations.

### Statistical analysis

Summary descriptive statistics were computed using proportions for categorical variables and mean ± standard deviation for continuous variables. The Pearson chi-square test or the Fisher exact test was used for categorical values as appropriate. Normality was assessed using the Shapiro–Wilk test. Either the Student *t* test or the Mann–Whitney *U* test was used for continuous variables, as appropriate.

We performed a pairwise comparison analysis to determine the association between perioperative protein expression patterns and the severity of PGD. PGD severity scores (1 to 3) determined at T0, T24, T48, and T72 h were used to define three comparisons: 2 versus 1, 3 versus 1, and 3 versus 2 for each timepoint. This resulted in a total of 12 possible comparisons. Within each comparison, differential expression of plasma proteins expressed in log2 (data + 1) scale was determined using Bayesian adjusted t-statistics as implemented in the linear models for microarray data (LIMMA) R package^18^. A multiple hypothesis testing correction was performed for each comparison using the Benjamini–Hochberg method^19^. Proteins were differentially expressed between PGD severity score levels if the false detection rate (FDR)-adjusted p-value was less than 0.25. Log2 fold changes for significant proteins were plotted using GraphPad Prism version 9.2.

We performed a sensitivity analysis to determine the association between biomarker expression patterns and development of PGD3 at T48–72 h, the most severe and persistent form of PGD. For this, we selected proteins whose levels correlated with severity of PGD in at least 3 of the 12 pairwise comparisons in the prior analysis. We compared their expression levels in patients who did or did not develop PGD3 at T48–72 h using LIMMA with significance achieved at an FDR-adjusted p-value < 0.10. We applied multiple hypothesis testing correction using the Benjamini–Hochberg method.

We performed a temporal analysis to explore the association between protein evolution patterns in patients who did or did not develop PGD3 at T48–72 h. For this analysis, we fit a linear mixed effect model (LMM) for each biomarker level. Because time and biomarker level are not linearly associated, B-spline basis on time, ${f}_{B}\left(time\right)$, was used to induce nonlinear structure. Moreover, ${f}_{B}\left(time\right)$, PGD3, and ${f}_{B}\left(time\right)\times$ PGD3 were used as fixed effects, and random effects were allowed across subjects. ${f}_{B}\left(time\right)\times$ PGD3 captures whether biomarkers are differentially expressed due to time and PGD status. The LMMs were fitted on a log scale when there were no missing data to reduce residual errors.

Overlap weighting^20–22^ was used to adjust for the following patient characteristics and three operative factors: BMI, hypertension, type of transplant (single versus bilateral), ex vivo lung perfusion (EVLP), and type of intraoperative extracorporeal life support (ECLS). We used overlap weighting here to achieve exact balance in case of any confounders between PGD and non-PGD groups. Analysis of variance p-values for testing whether the LMM coefficient of ${f}_{B}\left(time\right)\times$ PGD3 is zero were adjusted using the Benjamini–Hochberg procedure for the full and overlap weighted cohorts, respectively. LMM coefficient of ${f}_{B}\left(time\right)\times$ PGD3 close to 0 suggests that cytokine evolutions are significantly different between patients who did or did not develop PGD3 at T48–72 h. Statistical analyses were conducted using R. GraphPad and R were used to create figures. A two-sided p-value < 0.05 was considered significant.

### Ethics approval and consent to participate

This study was approved by the Institutional Review Board (IRB) for Human Subject Research for Baylor College of Medicine (IRB number: H-42256).

## Results

### Study population

Demographics and clinical characteristics of recipients and donors (n = 40) are summarized in Table 1. Of the 40 patients included in the study, 22 (55%) had PGD3 at some point after transplant from T0 to T72 h; 12 (30%) patients were diagnosed with PGD3 at T48–72 h. Characteristics associated with a higher risk of PGD3 at T48–72 h included a larger body mass index, a greater prevalence of systemic hypertension, and the intraoperative use of ECLS. As expected, these patients had worse clinical outcomes, although statistical significance was not reached for several variables likely due to sample size. No patient developed hyperacute graft rejection within 72 h.

**Table 1:** Demographics and clinical characteristics of 40 lung transplant recipients and donors composing the study cohort.

Variable	Total N = 40	( −) PGD3 at T48–72 h n = 28 (70%)	( +) PGD3 at T48–72 h n = 12 (30%)	P value
**Recipient characteristics**				
Women	12 (30%)	9 (32.14%)	3 (25%)	0.736
Age (years)	51.85 ± 14.77	50.21 ± 15.31	55.67 ± 13.24	0.291
Race				0.294
White	34 (85%)	25 (89.29%)	9 (75%)	
African American	5 (12.50%)	2 (7.14%)	3 (25%)	
Other	1 (2.50%)	1 (3.57%)	0 (0%)	
BMI (kg/m^2^)	26.05 ± 5.39	24.73 ± 5.10	29.17 ± 4.91	0.015
Primary disease				0.372
Restrictive lung disease	20 (50%)	12 (42.86%)	8 (66.67%)	
Cystic fibrosis	10 (25%)	9 (32.14%)	1 (8.33%)	
COPD	9 (22.50%)	6 (21.48%)	3 (25%)	
Pulmonary vascular disease	1 (2.50%)	1 (3.57%)	0 (0%)	
Diabetes	13 (32.50%)	12 (42.86%)	1 (8.33%)	0.063
Hypertension	16 (40%)	8 (28.57%)	8 (66.67%)	0.037
History of smoking*	21 (52.50%)	14 (50%)	7 (58.33%)	0.736
LAS	43.07 ± 11.76	42.83 ± 10.70	43.62 ± 14.45	0.849
Baseline creatinine (mg/dL)	0.97 ± 0.75	1.0 ± 0.89	0.88 ± 0.19	0.630
Condition at time of transplant				1.000
Not hospitalized	37 (92.50%)	26 (92.86%)	11 (91.67%)	
In ICU	3 (7.50%)	2 (7.14%)	1 (8.33%)	
Life support before transplant^†^	4 (10%)	3 (10.71%)	1 (8.33%)	1.000
Mean pulmonary artery pressure (mPAP)	26.85 ± 9.28	25.21 ± 9.15	30.67 ± 8.77	0.089
mPAP > 20 mmHg	30 (75%)	20 (71.43%)	10 (83.3%)	0.693
Prior thoracic surgery (non-transplant)	5 (12.50%)	3 (10.71%)	2 (16.67%)	0.627
Prior pleurodesis	1 (2.50%)	1 (3.57%)	0 (0%)	1.000
**Donor characteristics**				
Age (years)	38.5 ± 13.17	36.75 ± 12.76	42.58 ± 13.76	0.203
Women	12 (30%)	9 (32.14%)	3 (25%)	0.725
Extended criteria donor^‡^	19 (47.50%)	14 (50%)	5 (41.67%)	0.629
Diabetes	4 (10%)	3 (10.71%)	1 (8.33%)	1.000
Hypertension	18 (45%)	11 (39.29%)	7 (58.33%)	0.315
Smoker ever	22 (55.0%)	17 (60.71%)	5 (41.67%)	0.315
Smoker > 20PYH	3 (7.50%)	3 (10.71%)	0 (0%)	0.541
Sex mismatch	8 (20%)	6 (21.43%)	2 (16.67%)	1.0
Donor type DCD	3 (7.50%)	2 (7.14%)	1 (8.33%)	1.000
Ex-vivo lung perfusion^§^	11 (27.50%)	7 (25%)	4 (33.33%)	0.704
**Operative characteristics**				
Type of transplant bilateral	31 (77.50%)	20 (71.43%)	11 (91.67%)	0.233
Intraoperative support ECLS	25 (62.50%)	14 (50%)	11 (91.67%)	0.015
Total ischemic time (min)	409.55 ± 168.11	389.71 ± 165.52	455.83 ± 172.10	0.260
**Outcomes**				
Postop length of stay (days)	19.90 ± 20.45	17.07 ± 15.15	26.50 ± 29.18	0.185
Peak lactate within 72 h (mg/dL)	7.68 ± 4.15	6.97 ± 3.96	9.33 ± 4.26	0.100
Post-op ECMO	6 (15%)	2 (7.14%)	4 (33.33%)	0.055
Mechanical ventilation ≥ 5 days	6 (15%)	2 (7.14%)	4 (33.33%)	0.055
Reintubated	5 (12.50%)	4 (14.29%)	1 (8.33%)	1.000
Tracheostomy	7 (17.50%)	3 (10.71%)	4 (33.33%)	0.168
90-day mortality	2 (5%)	1 (3.57%)	1 (8.33%)	0.515
1-year mortality	4 (10%)	2 (7.14%)	2 (16.67%)	0.570

Values are n (%) or mean ± SD.

*Bilateral* double lung transplant, *BMI* body mass index, *COPD* chronic obstructive pulmonary disease, *DCD* donor after cardiac death, *ECLS* extracorporeal life support, *ECMO* extracorporeal membrane oxygenation, *ICU* intensive care unit, *LAS* lung allocation score, *mPAP* mean pulmonary arterial pressure, *Multi-organ* double lung and additional organs, *PGD* primary graft dysfunction, *Postop* postoperative, *SD* standard deviation, *Single* single lung transplant, *20PYH* 20 pack-year history of smoking.

*Two patients had used smokeless tobacco (snuff).

^†^Life support before transplant included ventilator or non-invasive positive pressure vent.

^‡^One or more of the following: age > 55 years, anticipated ischemia > 6 h, DCD, PaO_2_/FiO_2_ < 300, donor > 20PYH smoker.

^§^Using portable ex-vivo lung perfusion system.

### Pairwise comparison analysis of protein expression patterns and severity of PGD

**Figure 1:**
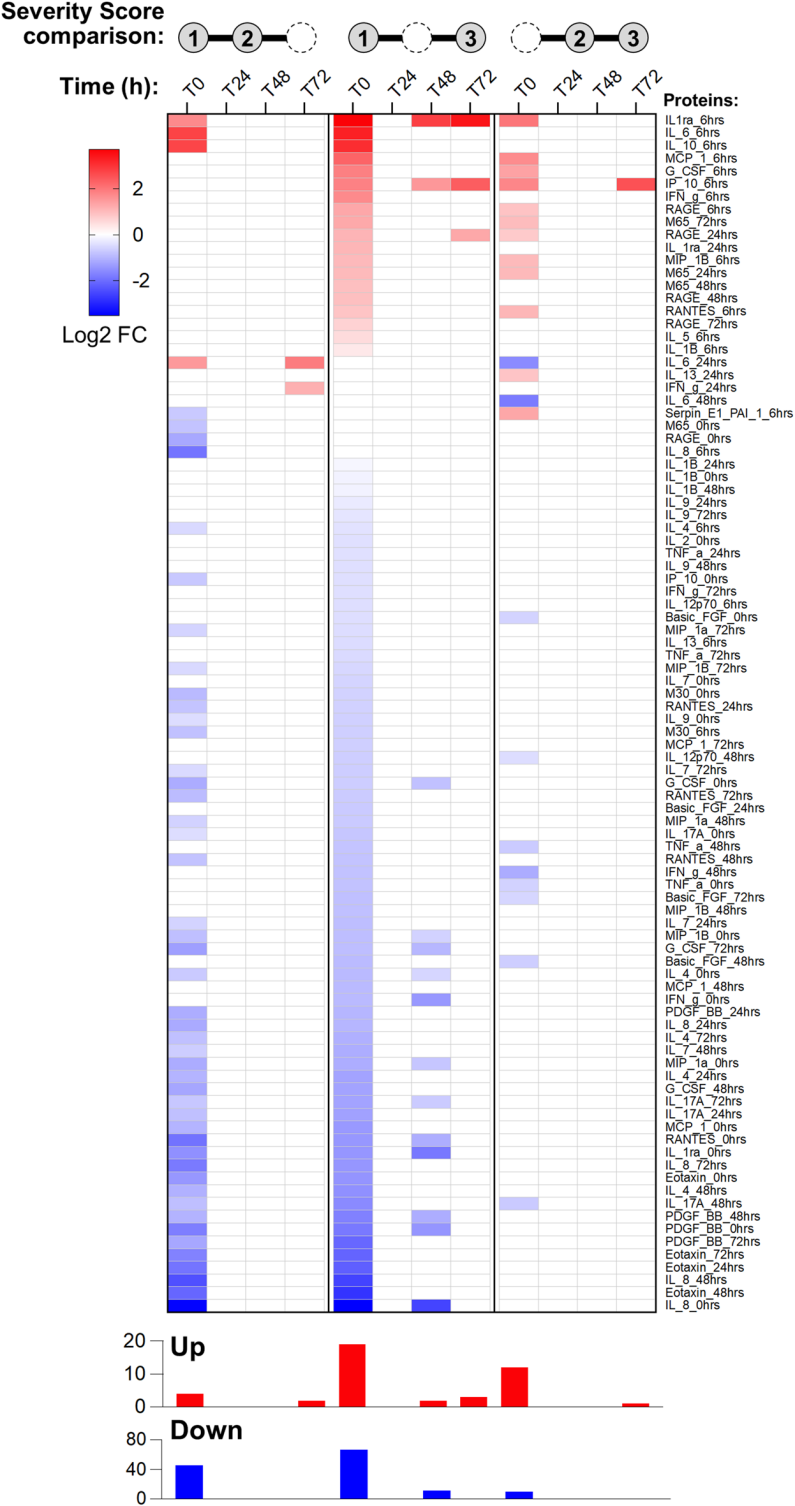
Pairwise comparison analysis. We performed a comprehensive differential protein analysis for pairs of PGD score levels (1, 2, and 3) at each of the individual time points from T0–T72 h. Per convention, T0 refers to the 6-h time point post-reperfusion. Summaries of upregulated and downregulated cytokines for each PGD level pairwise comparison and each time point are presented as barcharts. Individual proteins and the time point at which each was measured are listed on the right-hand side of the table. 0 h refers to pretransplant. 6 h refers to T0 or 6 h post-transplant reperfusion; 24, 48, and 72 h refer to 24, 48, and 72 h post-transplant reperfusion, respectively. The image was created using GraphPad Prism version 9.2 (https://www.graphpad.com/updates/prism-920-release-notes).

We used the annotated PGD severity scores, 1 to 3, to set up pairwise comparisons between patients with different levels of PGD severity at each post-transplant time point (T0–T72 h). Using a threshold FDR-adjusted p-value < 0.25, we identified multiple diverse differences in protein expression profiles associated with severity of PGD across multiple comparisons (Fig. 1). A robust signature for PGD3 versus PGD1 was observed at T0 and T48 h. Notably, IP10 and interleukin-1 receptor antagonist (IL-1Ra) were upregulated at 6 h post-lung transplant reperfusion in 5 of the 12 comparisons.

### Sensitivity analysis of protein expression patterns associated with PGD3 at T48–72 h

**Figure 2:**
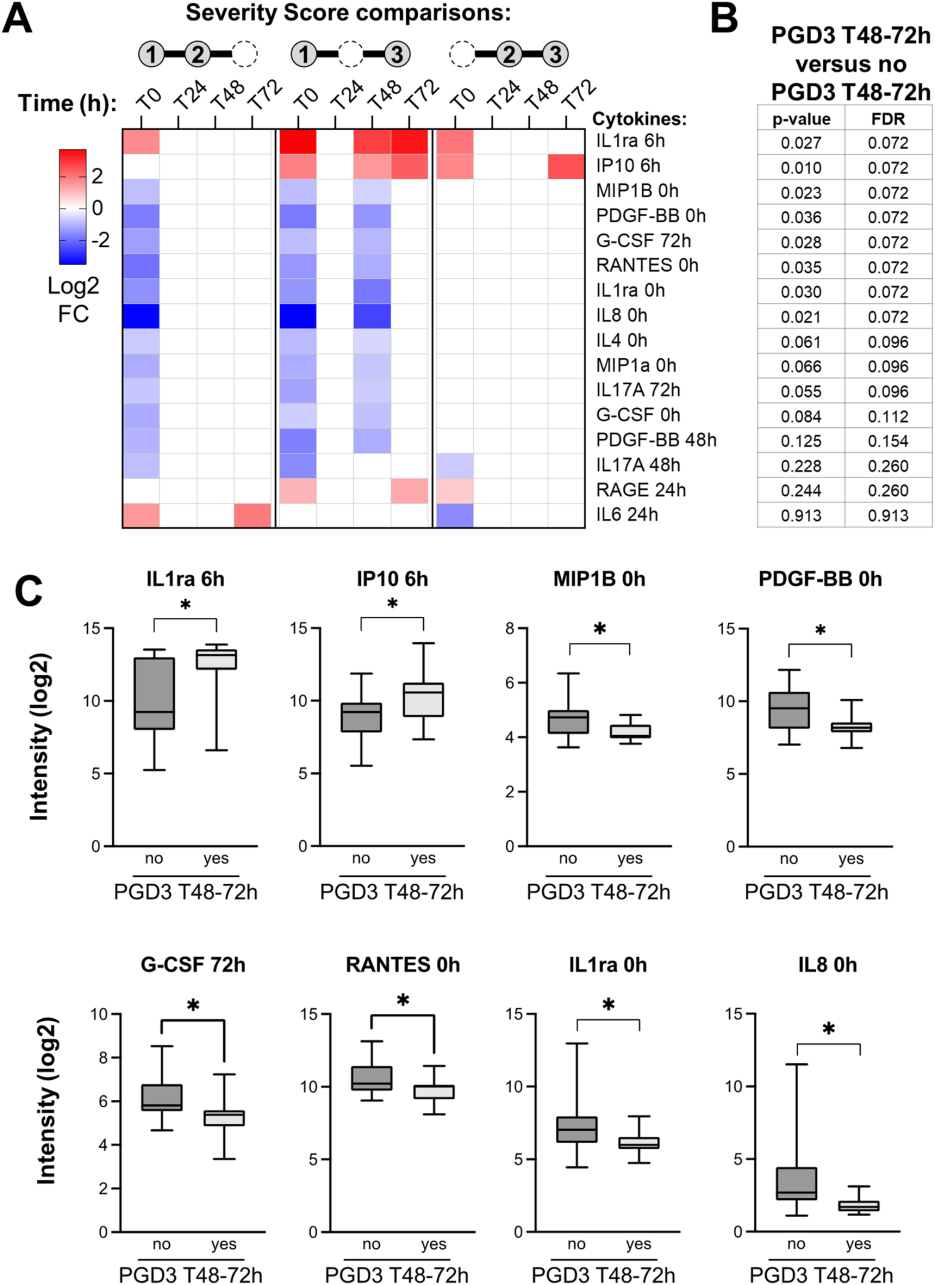
Sensitivity analysis. This analysis was performed to determine the effect of differential protein expression patterns on the development of PGD3 at T48–72 h. (**A**) We selected 16 protein expression patterns from the pairwise comparison analysis that reached significance in at least 3 of the 12 comparisons at FDR-adjusted p < 0.25. (**B**) We analyzed whether these 16 expression patterns were significantly different between patients who developed PGD3 at T48–72 h. Eight of the 16 protein expression patterns were associated with PGD3 at T48–72 h at p < 0.05 and 11 of 16 at FDR-adjusted p < 0.1. (**C**) Boxplots for selected cytokines associated with PGD3 at T48–72 h at p < 0.05. *p < 0.05. The image was created using GraphPad Prism version 9.2 (https://www.graphpad.com/updates/prism-920-release-notes).

We selected 16 protein expression patterns associated with the severity of PGD in at least 3 of the 12 comparisons from the previous analysis (Fig. 2A). Using a p-value < 0.05, 8 of these 16 protein expression patterns were associated with patients who developed PGD3 at T48–72 h, including the following patterns (Fig. 2B,C): (1) downregulation of IL-1Ra, macrophage inflammatory protein (MIP)-1beta, platelet derived growth factor (PDGF)-BB, RANTES, and IL-8 before transplant; (2) upregulation of IL-1Ra and IP-10 at 6 h post-transplant; and (3) upregulation of granulocyte colony-stimulating factor (G-CSF) at 72 h post-transplant. Using a threshold FDR adjusted p-value < 0.1, we detected an additional 3 biomarker expression patterns associated with patients who developed PGD3 at T48–72 h, including the following: (1) downregulation of IL-4 and MIP-1A before transplant and (2) downregulation of IL-17 at 72 h post-transplant (Fig. 2B).

### Temporal analysis of protein evolution patterns associated with PGD3 at T48–72 h

**Figure 3:**
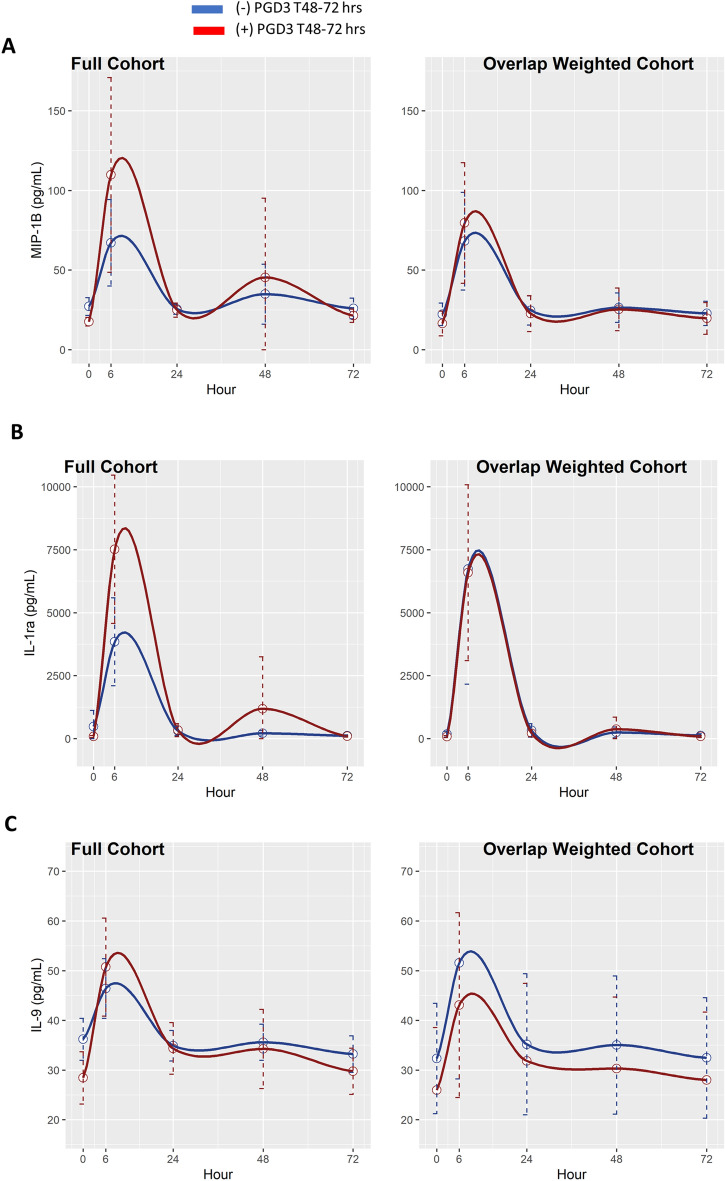
Temporal analysis. Differences in evolution for circulating biomarkers in patients with (red) or without (blue) PGD3 at T48–72 h in the full and overlap weighted cohort are shown. The overlap weighted cohort was adjusted for the following factors: BMI, hypertension, type of transplant, EVLP, and type of ECLS. (**A**) Macrophage inflammatory protein (MIP)-1B, (**B**) interleukin (IL)-9, and (**C**) interleukin-1 receptor antagonist (IL-1Ra). Circles represent the average biomarker level at respective time points; dotted lines represent 95% confidence intervals for the biomarker level at respective time points. The image was created using R software version number 4.1.3 (https://cran.r-project.org).

**Figure 4:**
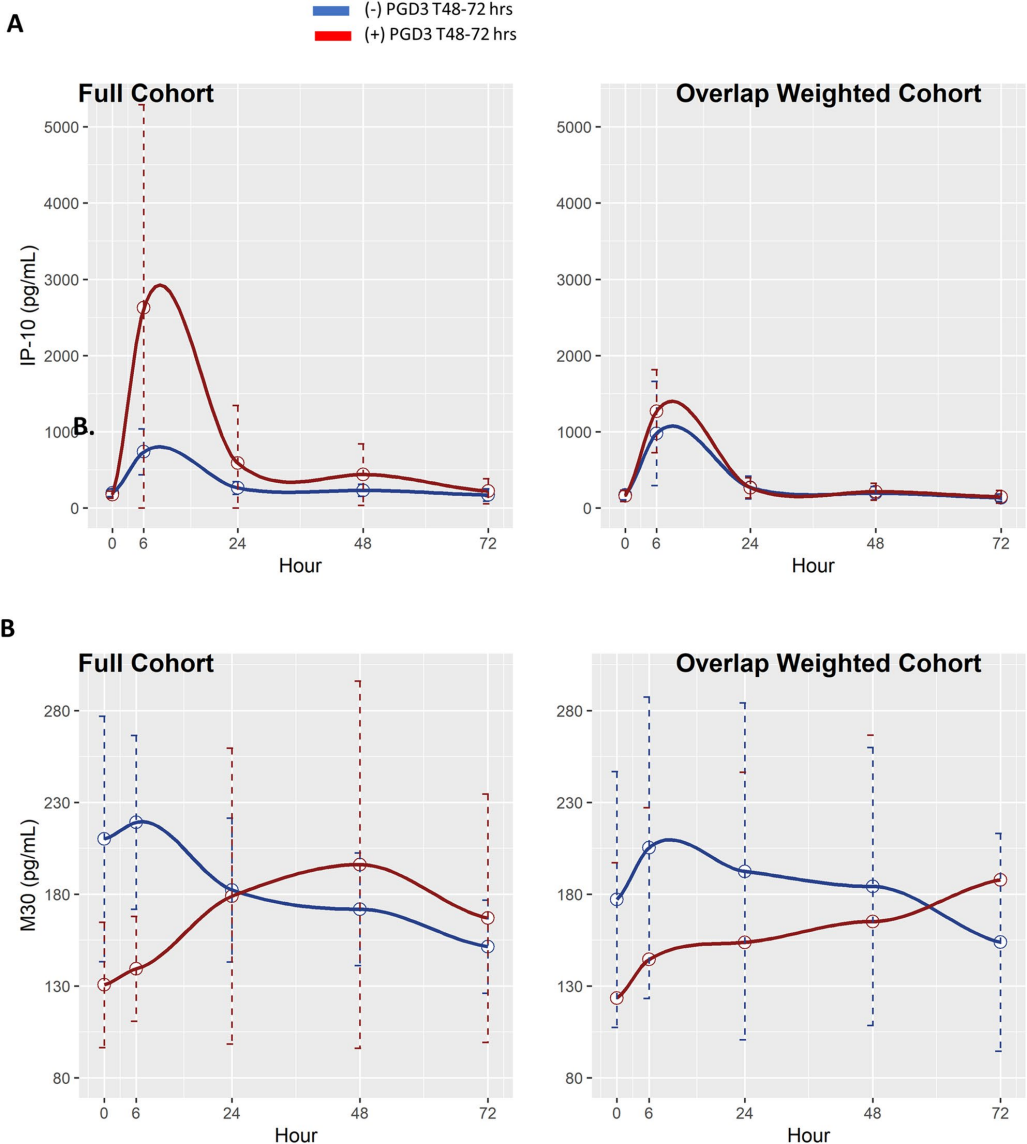
Temporal analysis. Differences in evolution for circulating biomarkers in patients with (red) or without (blue) PGD3 at T48–72 h in the full and overlap weighted cohort are shown. The overlap weighted cohort was adjusted for the following factors: BMI, hypertension, type of transplant, EVLP, and type of ECLS. (**A**) Interferon γ-induced protein (IP)-10 and (**B**) M30. Circles represent the average biomarker level at respective time points; dotted lines represent 95% confidence intervals for the biomarker level at respective time points. The image was created using R software version number 4.1.3 (https://cran.r-project.org).

We examined the differences in the temporal expression of circulating plasma proteins over 72 h post-reperfusion between patients who did or did not develop PGD3 at T48–72 h. For this analysis, we used LMM and overlap weighting adjusted for BMI, hypertension, use of ECLS, type of transplant (single versus bilateral), and use of EVLP. Statistical differences were noted for MIP-1B, IL-1Ra, IL-9, IP-10, and M30 before adjusting for multiple testing in both the non-overlap weighting and overlap weighting cohorts (Table 2, Figs. 3, 4). After adjusting for multiple testing, changes over time in IP-10 and M30 remained significant in the non-overlap weighted cohort, but not in the overlap weighted cohort (Table 2, Fig. 4). This suggests that the differences in IP-10 and M30 changes over time seen in patients who did or did not develop PGD3 at T48–72 h may have been affected by BMI, hypertension, and operative factors such as use of ECLS, type of transplant, and EVLP.

**Table 2:** Biomarker evolution over 72-h post-lung transplant between patients with and without PGD3 at T48–72 h.

Biomarker	Non-overlap weighted	Overlap weighted
MIP-1${\varvec{\upbeta}}$	0.0251 (0.1255)	0.0236 (0.1473)
IL-6	0.4239 (0.6887)	0.3744 (0.6512)
IFN-γ	0.1334 (0.4040)	0.3254 (0.6512)
IL-1Ra	0.0062 (0.0519)	0.0075 (0.0940)
TNF-α	0.0726 (0.2592)	0.0695 (0.2481)
RANTES	0.0598 (0.2491)	0.0572 (0.2385)
IL-2	0.4854 (0.6887)	0.4255 (0.6512)
IL-1B	0.7725 (0.8779)	0.7618 (0.8656)
Eotaxin	0.8823 (0.9590)	0.8727 (0.9486)
Basic-FGF	0.4529 (0.6887)	0.4428 (0.6512)
PDGF-BB	0.4640 (0.6887)	0.2761 (0.6512)
IL-9	0.0177 (0.1109)	0.0170 (0.1418)
IP-10	0.0010 (0.0253)	0.0429 (0.2146)
IL-13	0.7115 (0.8471)	0.6089 (0.7249)
MCP-1	0.9717 (0.9960)	0.9712 (0.9944)
IL-8	0.5234 (0.6887)	0.5164 (0.6794)
MIP-1${\upalpha }$	0.1786 (0.4466)	0.1654 (0.4594)
G-CSF	0.4318 (0.6887)	0.4205 (0.6512)
IL-7	0.9960 (0.9960)	0.9944 (0.9944)
IL-12p70	0.4841 (0.6887)	0.4398 (0.6512)
IL-17A	0.5056 (0.6887)	0.5029 (0.6794)
RAGE	0.1454 (0.4040)	0.1577 (0.4594)
PAI-1	0.5910 (0.7388)	0.5991 (0.7249)
M30	0.0021 (0.0263)	0.0023 (0.0578)
M65	0.3813 (0.6887)	0.3801 (0.6512)

Because time and cytokine level are not linearly associated, B-spline basis on time, ${f}_{B}\left(time\right)$, was used to induce nonlinear structure. Moreover, ${f}_{B}\left(time\right)$, PGD3, and ${f}_{B}\left(time\right)\times$ PGD3 were used as fixed effects, and random effects were allowed across subjects. ${f}_{B}\left(time\right)\times$ PGD3 captures whether cytokines are differently expressed due to time and PGD status. The LMMs were fitted on log scale when there were no missing data to reduce residual errors. Overlap weighting was also used to adjust for the following five factors: BMI, hypertension, type of transplant (single versus bilateral), EVLP, and type of intraoperative ECLS. We used overlap weighting here to achieve exact balance in means of any confounders between patients that did or did not develop PGD3 at T48–72 h. *p-*value less than 0.05 suggests that the biomarker evolution is different between patients that did or did not develop PGD3 at T48–72 h. Values in the parentheses are the Benjamin-Hochberg adjusted *p-*values for multiple comparison testing.

*ECLS* extracorporeal life support, *EVLP* ex-vivo lung perfusion, *FGF* fibroblast growth factor, *G-CSF* granulocyte colony-stimulating factor, *IL-1Ra* interleukin-1 receptor antagonist, *IP-10* interferon gamma-induced protein 10, *MCP-1* monocyte chemoattractant protein-1, *PAI-1* plasminogen activator inhibitor-1, *PDGF* platelet derived growth factor, *TNF* tumor necrosis factor.

## Discussion

Biomarkers for PGD are mediators and byproducts of the molecular events that characterize the pathogenesis of the disease. Here, we have identified clusters of cytokines, chemokines, growth factors, and apoptotic proteins strongly associated with the clinical grade and duration of PGD.

Deciding which of these biomarkers to use in clinical practice is challenging. Ideally, the biomarker would correlate strongly with PGD across all analyses. In this regard, IP-10 would be a good candidate. IP-10 levels at 6 h post-lung transplant reperfusion correlated with the severity of PGD in 5 of the 12 pairwise comparisons. Moreover, IP-10 levels at 6 h correlated with the development of PGD3 at T48–72 h in the sensitivity analysis and the temporal analysis, except when adjusted for multiple testing in the overlap-weighted cohort. Thus, IP-10 appears to be a strong candidate for use as a biomarker for PGD severity and duration, although its temporal trends could be affected by operative factors.

Given the complexity of the molecular events underlying PGD, it is unlikely that a single biomarker would be sufficient to detect its severity and duration. Thus, considering other important, although perhaps less robust, correlations observed in our analysis is worthwhile. MIP-1B and RANTES are two additional chemokines that correlated with PGD. MIP1-B expression profiles were significantly associated with PGD in the paired comparisons analysis and the sensitivity analysis. The temporal evolution of MIP1-B was associated with PGD3 at T48–72 h, except when adjusted for multiple testing. RANTES levels correlated with the severity of PGD in the paired analysis and the duration of PGD in the sensitivity analysis. Our findings and those of others suggest that chemokines could be important in the pathogenesis of PGD and may be reasonable candidates to use in a panel of biomarkers for PGD severity and duration^15,17,23^.

Furthermore, our results were consistent with those of Hashimoto and colleagues who showed elevated levels of M30 and M65 at T24–48 h post-lung transplant in patients with PGD3^11^. We observed a gradual and late rise in M30 levels associated with PGD3 at T48–72 h, suggesting a phased onset of apoptosis. M65, which is an indicator of both apoptosis and necrosis, did not correlate with PGD in our temporal analysis. However, the paired comparison analysis suggested that M65 levels had a delayed association with PGD severity.

In the current study, we found a consistent association between increased levels of IL-1Ra and the severity and duration of PGD. It is difficult to explain this association. IL-1Ra is an immune modulator that counteracts the effects of IL-1 alpha and beta. Whether the elevated levels result in exacerbation of PGD or are a byproduct of a reparative response is unclear. It is conceivable that increased IL-1Ra levels correspond to a depletion of IL-1. Although we did not detect differences in IL-1 levels in the temporal analysis, we did identify reduced levels of IL-1B at T24–48 h in the paired analysis in at least one comparison (T0 PGD3 versus 1). This supports the findings of Hoffman and colleagues who showed a precipitous reduction in IL-1B levels after reperfusion along with elevated levels of IL-1Ra in patients with severe PGD compared with those without PGD^15^. Thus, our findings and those of others support the use of IL-1Ra as a biomarker for PGD.

Several protein expression patterns in our study were notable for the lack of association with PGD, findings that contradict those of previous studies^14^. For example, Mathur and colleagues found increased levels of cytokines including IL-6, IL-8, IL-10, and TNF-α in patients who developed PGD. We did not find these same correlations, but we did note an increase in IL-10 at 6 h that was associated with PGD grade 3 rather than 1. We also found a decrease in IL-6, TNF-α, and IL-8 associated with the severity of PGD. Lower baseline levels of IL-8 were associated with PGD3 at T48–72 h (Fig. 2A,B). Although the association of IL-9 with severity of PGD was relatively weak, we found an indirect correlation between IL-9 levels and PGD severity (Fig. 1).

We also explored the role of markers of lung epithelial damage or endothelial dysfunction as biomarkers for PGD. Christie et al.^8^ showed that levels of sRAGE, a marker of lung epithelial cell injury, were elevated in patients with PGD. We found a correlation between sRAGE levels and the severity of PGD in one of 12 pairwise comparisons. Pretransplant samples showed lower levels of sRAGE in patients with greater PGD severity, which is consistent with findings reported by Daoud et al.^5^. sRAGE levels were increased in postoperative samples at T24–72 h in our study. Although these results support those of Christie et al.^8^, our findings were seen in a single column of the pairwise analysis and not in the sensitivity or temporal analyses. In a separate study, Christie et al.^16^ showed that PAI-1, a marker of endothelial dysfunction, was elevated in patients with PGD. In our study, PAI-1 levels were different in the pairwise comparison in a single column, only at the 6-h time point. Our PAI-1 finding is difficult to interpret; PAI-1 expression was downregulated when comparing PGD2 versus 1 at T0 and upregulated when comparing PGD3 versus 2 at T0. We found no significant differences in PAI-1 expression patterns in the sensitivity or the temporal analyses. This could be due to differences in collection protocols or the lack of power to detect differences in the expression of these biomarkers.

This study had several limitations that should be considered when interpreting and generalizing the data. Our sample size was limited, which could have affected our ability to detect differences in biomarker expression (type II error). Additionally, because of the small sample size and the variability of biomarker expression at each time point, we were able to estimate only to pointwise confidence intervals. However, we controlled for type I error by using appropriate statistical models, thus strengthening our identification of significant biomarkers. We believe that the in-depth data analysis in our small study provides important insight for future work on a larger scale.

The population in our study was not homogeneous, and operative factors such as use of ECLS, type of lung transplant (single versus double), and the use of EVLP could confound the interpretation of results. In fact, these weaknesses are among the reasons why biomarkers are not used heavily in clinical practice. It is generally cost prohibitive to obtain large samples sizes in homogeneous populations for studies designed to draw definitive conclusions. Moreover, the rates of PGD in this series were higher than those reported in large multicenter studies^24^, but the risk factors and outcomes associated with PGD were similar. The higher rates in our study may be due to the use of the updated 2016 ISHLT scoring guidelines, which increase detection of PGD, particularly in extubated patients^5^. However, our PGD rates were not entirely different from those in a recent international multicenter cohort^25^. Finally, caution should be taken when analyzing baseline biomarkers and their effects on PGD as we have previously reported; although potentially informative, these relationships can be heavily influenced by confounding variables^5^. The associations between downregulation of preoperative biomarkers and development of PGD more likely reflected the delta increase in biomarkers as evidenced by the temporal evolution analysis.

Nevertheless, our study has several strengths. This study of serial samples from 40 consecutive consented patients is one of the largest recent single-center experiences for PGD biomarker analysis in lung transplantation. Since the early biomarker studies from the LTOG consortium, the PGD scoring system has been revised to improve consistency and sensitivity^4,5^. Additionally, perioperative practices in lung transplantation have evolved, including greater use of ECLS and EVLP^25–27^. Although these perioperative practices could confound our results, it is almost impossible to study biomarkers associated with PGD in the current era without including them. Similarly, the use of postoperative extracorporeal membrane oxygenation could confound the interpretation of postoperative biomarkers; however, this is a common treatment for severe PGD and would also be difficult to exclude. Table 1 shows that only the mode of intraoperative support was statistically different between groups. In our experience, intraoperative extracorporeal life support is primarily used prophylactically at the start of the case, depending on the surgeon’s opinion as to whether it will facilitate the operation. It may also be used depending on the results of a short test clamp of the pulmonary artery or single-lung ventilation. Within the study period, only 1.77% of cases required conversion for urgent indications such as profound hypoxia, air, bleeding, or hemodynamic instability. It remains unresolved whether use of intraoperative ECMO alters the reperfusion inflammatory milieu in the lung allograft; this topic warrants additional investigation. Postoperative ECMO was not used for prophylactic indications in this series; therefore, all postoperative ECMO was graded as PGD3.

In addition, we used several innovative statistical methods to validate our findings. We utilized a novel pairwise comparison analysis with LIMMA to identify a range of possible molecular signatures for PGD^18^. Our sensitivity analysis helped reinforce the association between biomarkers and the duration of severe PGD. In a temporal analysis, we used overlap weighting to adjust for possible confounding factors^20–22^.

Detecting cytokines consistently can be challenging and often depends on factors such as freeze–thaw cycles, storage duration, and specimen processing^28^. We used a single freeze–thaw cycle and limited the storage duration. All samples were processed similarly in the Department of Regenerative Medicine at the Texas Heart Institute, which has individuals with significant expertise and experience in sample processing for cytokine and cell population analysis, including storing and processing samples for several national clinical trials^29^.

The discovery and application of biomarkers has revolutionized the treatment of patients with lung cancer, heart failure, and myocardial ischemia, but it has not yet been applied to the care of patients in whom complications develop after lung transplantation^30–32^. We propose that it is time to incorporate biomarker analysis into clinical practice in lung transplantation. Based on our analysis, IP-10, IL-1Ra, MIP-1B, PDGF-BB, RANTES, IL-8, G-CSF, and M30 are particularly strong candidates for biomarkers of PGD severity and duration. We recommend the clinical use and continued examination of a panel of biomarkers that could allow us to detect PGD early, predict its clinical course, monitor its progression, provide mechanistic insight for drug development, and establish benchmarks for therapeutic efficacy.

## Supplementary Information

Supplementary Table 1.

## Data Availability

The data that support the findings of this study are available on request from the corresponding author. The data are not publicly available due to privacy or ethical restrictions.

## Abbreviations

ECLS: Extracorporeal life support
ELISA: Enzyme-linked immunoassay
EVLP: Ex vivo lung perfusion
FDR: False detection rate
IR: Ischemia–reperfusion
IRB: Institutional Review Board
ISHLT: International Society for Heart and Lung Transplantation
LIMMA: Linear model for microarray data
LMM: Linear mixed effect model
LTOG: Lung Transplant Outcomes Group
PGD: Primary graft dysfunction
