# Supplementary material for: Plasma protein biomarkers for primary graft dysfunction after lung transplantation: a single-center cohort analysis
Source: Sci Rep. 2022 Sep 27;12:16137. doi: 10.1038/s41598-022-20085-y (PMC9515157; doi:10.1038/s41598-022-20085-y)
Supplement: Supplementary file 1 — Supplementary Table 1. [file 41598_2022_20085_MOESM1_ESM.docx]

**Supplementary Table 1**. Overview of cytokines assessed in this study

| **Cytokines** |
| --- |
| **Pro-inflammatory cytokines** |
| IL-6 |
| INF-γ |
| TNF-α |
| IL-17A |
| IL-1b |
| **Anti-inflammatory cytokines** |
| IL-10 |
| IL-4 |
| IL1-Ra |
| IL-13 |
| **Chemokines (innate)** |
| MIP-1A |
| MIP-1B |
| MCP-1 |
| RANTES |
| Eotaxin |
| IP-10 |
| IL-8 |
| **Signaling cytokines** |
| IL-2 |
| IL-7 |
| IL-9 |
| **T cell differentiation and stimulation** |
| IL-12p70 |
| IL-5 |
| GM-CSF |
| G-CSF |
| **Growth factors** |
| Basic FGF |
| PDGF-BB |
| VEGF |
| IL-15 |

DC, dendritic cells; FGF, fibroblast growth factor; GM-CSF, granulocyte-macrophage colony- stimulating factor; IL, interleukin; INF, interferon; IP, Interferon γ-induced protein; MCP, monocyte chemoattractant protein; MIP, macrophage inflammatory protein; PDGF-BB, platelet-derived growth factor-BB; VEGF, vascular endothelial growth factor.
